# Efficacy and cost-effectiveness of Internet-based selective eating disorder prevention: study protocol for a randomized controlled trial within the ProHEAD Consortium

**DOI:** 10.1186/s13063-018-3161-y

**Published:** 2019-01-30

**Authors:** Stephanie Bauer, Sally Bilić, Christina Reetz, Fikret Ozer, Katja Becker, Heike Eschenbeck, Michael Kaess, Christine Rummel-Kluge, Hans-Joachim Salize, Silke Diestelkamp, Markus Moessner, the ProHEAD Consortium, Michael Kaess, Stephanie Bauer, Rainer Thomasius, Christine Rummel-Kluge, Heike Eschenbeck, Hans-Joachim Salize, Katja Becker, Katja Bertsch, Sally Bilić, Romuald Brunner, Johannes Feldhege, Christina Gallinat, Sabine C. Herpertz, Julian Koenig, Sophia Lustig, Markus Moessner, Fikret Ozer, Peter Parzer, Franz Resch, Sabrina Ritter, Jens Spinner, Silke Diestelkamp, Kristina Wille, Sabrina Baldofski, Elisabeth Kohls, Lina-Jolien Peter, Vera Gillé, Hanna Hofmann, Laya Lehner, Elke Voss, Jens Pfeiffer, Alisa Samel

**Affiliations:** 1 Center for Psychotherapy Research, University Hospital Heidelberg, Bergheimerstr. 54, 69115 Heidelberg, Germany; 2 Department of Child and Adolescent Psychiatry, Psychosomatics and Psychotherapy, Philipps-University Marburg and Marburg Center for Mind, Brain and Behavior (MCMBB), Philipps-University Marburg, Marburg, Germany; 3 Department of Psychology, University of Education Schwäbisch Gmünd, Oberbettringer Str. 200, 73525 Schwäbisch Gmünd, Germany; 4 Section for Translational Psychobiology in Child and Adolescent Psychiatry, Department of Child and Adolescent Psychiatry, University Hospital Heidelberg, Blumenstraße 8, 69115 Heidelberg, Germany; 5 University Hospital of Child and Adolescent Psychiatry and Psychotherapy, University of Bern, Bolligenstr. 111, Stöckli, 3000 Bern 60, Switzerland; 6 Department of Psychiatry and Psychotherapy, Medical Faculty, Leipzig University, Semmelweisstraße 10, 04103 Leipzig, Germany; 7 Mental Health Services Research Group, Central Institute of Mental Health, Medical Faculty Mannheim/Heidelberg University, J5, 68159 Mannheim, Germany; 8 German Center for Addiction Research in Childhood and Adolescence, University Medical Center Hamburg-Eppendorf, Martinistr. 52, W29, 20246 Hamburg, Germany

**Keywords:** Eating disorders, Prevention, Internet, Dissonance, CBT, ProHEAD

## Abstract

**Background:**

The development of efficacious, cost-effective, and widely accessible programs for the prevention of eating disorders (EDs) is crucial in order to reduce the ED-related burden of illness. Programs using dissonance-based and cognitive behavioral approaches are most effective for the selective prevention of ED. Internet-based delivery is assumed to maximize the reach and impact of preventive efforts. However, the current evidence for Internet-based ED prevention is limited. The present trial evaluates the efficacy and cost-effectiveness of two new interventions (based on dissonance theory and principles of cognitive behavioral therapy (CBT)) that are implemented as add-ons to the existing Internet-based ED prevention program ProYouth.

**Methods:**

The trial is one of five sub-projects of the German multicenter consortium ProHEAD. It is a three-arm, parallel, randomized controlled superiority trial. Participants will be randomized to (1) the online program ProYouth (active control condition) or (2) ProYouth plus a structured dissonance-based module or (3) ProYouth plus a CBT-based chat group intervention. As part of ProHEAD, a representative school-based sample of *N* = 15,000 students (≥ 12 years) will be screened for mental health problems. *N* = 309 participants at risk for ED (assessed with the Weight Concerns Scale (WCS) and the Short Evaluation of Eating Disorders (SEED)) will be included in the present trial. Online assessments will be conducted at baseline, at end of intervention (6 weeks), at 6 months follow-up, and — as part of ProHEAD — at 12 and 24 months follow-up. The primary outcome is ED-related impairment (assessed with the Child version of the Eating Disorder Examination-Questionnaire (ChEDE-Q)) at the end of the intervention. Secondary outcomes include ED-related symptomatology at follow-up, ED-related stigma, ED-related help-seeking, and acceptance of and compliance with the interventions. For the health economic evaluation data on costs of the interventions, healthcare utilization and health-related quality of life will be assessed.

**Discussion:**

This is the first study augmenting a flexible prevention approach such as ProYouth with structured evidence-based modules in order to overcome some of the key limitations in the current practice of ED prevention.

**Trial registration:**

German Clinical Trials Register (DRKS), DRKS00014679. Registered on 25 April 2018.

**Electronic supplementary material:**

The online version of this article (10.1186/s13063-018-3161-y) contains supplementary material, which is available to authorized users.

## Background

Eating disorders (EDs) such as anorexia nervosa, bulimia nervosa, and binge eating disorder are severe mental illnesses associated with a substantial burden of disease and significant healthcare costs [1, 2]. Mortality is increased in all EDs and is highest among all mental disorders in the case of anorexia nervosa. EDs mainly develop during adolescence and young adulthood [3, 4] and affect both males and females. The prevalence of full and sub-threshold EDs is estimated at 15% in young women and 3% in young men [5]. In addition, many young people report ED-related attitudes and behaviors without meeting full diagnostic criteria. EDs are associated with high psychological and physical impairment and can also affect many other areas of young people’s lives, e.g., by leading to social isolation, reduced academic and professional performances, and an overall loss of quality of life [2].

Despite significant progress in prevention and treatment research over the past decades, current healthcare may not substantially alleviate the burden related to ED at the population level due to limited efficacy, availability, and reach of evidence-based interventions [6, 7]. While effective interventions for the various forms of ED exist, only a minority of patients actually benefit and recover. A major challenge in the treatment of ED is that less than 25% of those affected by ED actually seek and receive professional help. In many cases there is a substantial delay between symptom onset and access to care, leading to an increased risk of chronicity [8]. A recent systematic review revealed that help-seeking for ED is hindered by barriers such as stigma and shame, denial of and failure to perceive the severity of illness, negative attitudes towards seeking help, lack of encouragement from others to seek help, and limited knowledge about treatment resources [9]. There is consensus that it is of great importance to develop and disseminate evidence-based, cost-effective, and widely accessible prevention and early intervention tools for EDs, especially considering their high burden of illness, their challenging treatment, and their associated costs on both individual and societal levels.

Concerning the empirical evidence of ED prevention programs, three relevant systematic reviews and meta-analyses have been conducted. Stice and colleagues analyzed efficacy trials on 51 ED prevention programs. They identified a significant reduction of at least one established ED risk factor for 26 interventions and a reduction of current or future ED symptomatology for 15 interventions [10]. Selective programs proved to be more effective than universal programs, as well as programs with interactive components compared to more didactic interventions. Furthermore, multi-session programs showed larger intervention effects than single-session programs, as well as programs conducted by external facilitators compared to interventions delivered by school personnel. Although the findings were considered overall encouraging, most significant intervention effects were small, and many studies showed major methodological flaws [10].

Two more recent systematic and meta-analytic reviews independently summarized the effects of interventions for universal, selective, and indicated prevention of ED [11, 12]. Both reviews reported that the majority of interventions focused on selective prevention. Also both reviews identified the strongest evidence base for this type of preventive effort, i.e., interventions addressing individuals at risk for the development of an ED. In particular, two approaches were associated with a significant reduction of ED-specific risk factors and ED-related impairment: dissonance-based interventions and interventions based on the principles of cognitive behavioral therapy (CBT).

However, a number of remaining challenges were identified in these reviews (of which several will be addressed in the present trial). First, the content, intensity, and delivery mode of the interventions varied substantially, and comparisons to active control conditions are scarce, so that little is known about the relative efficacy of different interventions. Second, studies mostly included female adolescents and young adults (≥ 15 years), so that more research in younger samples and males is needed. Third, there is a need for research on how to improve uptake of and compliance with ED prevention among individuals at risk. Fourth, there is limited empirical knowledge on the long-term effects, cost-effectiveness, implementation, and dissemination of evidence-based prevention programs [11].

In order to enhance the reach of preventive efforts, an increasing number of Internet-based ED prevention programs have been developed in recent years. Such programs benefit from permanent availability and accessibility independent of time and location. They are easily accessible, they may be accessed anonymously, and they enable providers to tailor content to the needs of specific target groups. Several reviews have summarized the available evidence on Internet-based interventions in the field of ED. Overall, these reviews concluded that such programs are feasible and promising tools for prevention, self-help, treatment, and relapse prevention in ED [13–17]. However, they also stated that technology-enhanced care in ED is still an understudied area, that there are few adequately powered efficacy trials (especially in the field of prevention), and that there is hardly any information on the costs of interventions and their cost-effectiveness.

One of the more widely studied Internet-based prevention programs is ProYouth, which emerged from the previous programs Essprit [18] and YoungEssprit [19]. The program comprises several components that participants may use depending on their individual needs and preferences; i.e., ProYouth is a flexible (versus structured/manualized) intervention (see subsequent sections for a description of the components). Following pilot studies on the feasibility and acceptability of the program [18, 20], its efficacy was investigated in a large high-school sample in Germany [19]. An enhanced version of the program was subsequently implemented as part of the European Union (EU)-funded ProYouth initiative in several European countries. In this context, costs associated with the maintenance of the intervention [21] as well as the cost-effectiveness of different dissemination strategies were analyzed [22]. Furthermore, there is initial evidence that ProYouth may positively impact participants’ help-seeking behavior [23]. However, research also showed that ProYouth is mainly used by severely impaired participants (i.e., a “clinical” group) and that the actual target population — participants at risk for an ED or those with sub-threshold ED symptoms — rarely use the available modules (Bauer, Kindermann, Ozer, Moessner: Dissemination of an Internet-based Program for Prevention and Early Intervention: Relationship between Access Paths, User characteristics, and Program Utilization, submitted). Thus, there is a clear need to augment the program to further engage and benefit individuals at risk for the development of an ED. Therefore, the present trial focuses on the development and evaluation of two new intervention modules as add-ons to the existing ProYouth program.

## Rationale and objectives

The overall objective of the present trial is to enhance the potential of ED prevention in children and adolescents (C&A) with an elevated risk for the development of an ED. Therefore, based on the latest scientific evidence outlined above, two new intervention modules will be added to the existing Internet-based ED prevention program ProYouth.

The first module will follow a dissonance-based ED prevention approach that was introduced in the context of the program with the best empirical evidence to date, i.e., the Body Project [24]. The Body Project is a group intervention to reduce thin-ideal internalization and subsequent ED symptoms using an approach based on cognitive dissonance assuming that adolescents and young adults develop body dissatisfaction and weight/shape concerns because of their internalization of the thin ideal (i.e., extremely thin women are considered most attractive/beautiful in Western societies). The rationale for the intervention is that participants who engage in exercises questioning this ideal should benefit from the program. Questioning the thin ideal thereby would reduce thin-ideal internalization, which should subsequently lead to a reduction of ED risk factors (e.g., body dissatisfaction, weight/shape concerns, dieting, and negative affect) and symptoms. The efficacy of the Body Project in terms of a reduction of ED risk factors, symptoms, and illness onset was shown in a number of large-scale randomized controlled trials (RCTs) (e.g., [24]). More recently, Internet-based versions of dissonance-based interventions have been introduced [25, 26].

The second module to be evaluated in this trial is based on a CBT approach. Several programs for selective ED prevention are based on CBT principles (see [11] for an overview), such as the well-studied program Student Bodies [27]. For the present trial, a clinician-guided group-based approach will be used. Promising findings have been reported both for the face-to-face as well as for an online chat-based delivery of such an approach in samples of Australian women with high levels of body dissatisfaction. However, existing findings are preliminary due to small sample sizes [28].

Following the development of these two new intervention modules and their implementation within the existing Internet-based program ProYouth, the specific objectives of the present trial focus on the efficacy and health economic evaluation of these enhanced versions compared to ProYouth alone.

## Methods/design

### Design and setting

The present trial represents one of five sub-projects conducted within the German multicenter consortium ProHEAD (Promoting Help-seeking using E-technology for ADolescents). Collaborators at five sites join forces in order to recruit a large representative school-based sample (*N* = 15,000) as part of the central project (CP) of ProHEAD. The five sub-projects are nested within this CP.

The present study (sub-project 2 of the ProHEAD Consortium) is a three-arm, parallel, randomized controlled superiority trial investigating the efficacy and cost-effectiveness of two new intervention modules for C&A at risk for the development of EDs in comparison to an active control intervention. The ethical committee of the Medical Faculty of Heidelberg University has approved the study procedures and design.

### Participants

Male and female C&A aged 12 years or older (grades 6–13) are invited to participate in the school-based ProHEAD screening. Those who provide informed consent (student and at least one legal guardian) and complete the screening will be assigned to one of the five ProHEAD sub-projects depending on their screening results (sub-project 1: clinically relevant mental health problems [29]; sub-project 2: risk for ED; sub-project 3: risky alcohol use [30]; sub-project 4: risk for depression [31]; sub-project 5: no clinically relevant mental health problems [32]).

Eligibility criteria are checked as part of the screening procedure. Inclusion criteria for the present trial are sufficient German language skills, Internet access, a high risk for an ED (Weight Concerns Scale (WCS; score > 57 [33, 34]) or sub-threshold ED symptoms based on the Short Evaluation of Eating Disorders (SEED [35]; bingeing and/or vomiting at least once a week). Individuals are excluded if they meet criteria for clinically relevant impairment as defined for sub-project 1 of ProHEAD [29]. This means that exclusion criteria for the present trial are severe ED symptoms, indicated by a body mass index (BMI) below the 5th percentile (adjusted for age and gender) and concurrent fear of weight gain, or daily bingeing, or daily vomiting assessed with the SEED [35]. Additional exclusion criteria include current suicidality, high depressive impairment measured with the Patient Health Questionnaire-9 modified for Adolescents (PHQ-A; score > 9 [36, 37]), high alcohol consumption assessed with the Alcohol Use Disorders Identification Test (AUDIT; score ≥ 20 [38]), and clinically relevant general psychological impairment operationalized with the Strengths and Difficulties Questionnaire (SDQ Total score ≥ 20; SDQ Emotional Symptoms score > 6; SDQ Conduct Problems score > 4; SDQ Hyperactivity score > 6; SDQ Peer Relationship Problems score > 5 [39, 40]).

Criteria for the allocation of participants to the five individual ProHEAD randomized controlled trials (RCTs) are based on the latest scientific evidence. However, this is the first time that the overall algorithm screening for various mental health problems is simultaneously applied on a consortium-wide basis. Therefore, an intermediate data analysis will be conducted following completion of 10% of the screening assessments (*N* = 1500) in order to determine the actual allocation ratio to the five ProHEAD trials and to adjust the screening algorithm if necessary.

### Recruitment and study procedures

The ProHEAD Consortium will recruit a school-based sample of 15,000 C&A aged 12 or older in grades 6–13 in five regions of Germany (Hamburg, Heidelberg, Leipzig, Marburg, Schwäbisch Gmünd). The procedures with respect to selection of schools are described in the study protocol of sub-project 1 of the ProHEAD Consortium [29].

Written informed consent will be obtained from C&A and their legal guardians prior to inclusion into the project. As part of the ProHEAD CP, assessments of sociodemographic information, healthcare utilization, help-seeking, and a broad variety of mental health problems and health-risk behaviors will be included in the screening and the two annual follow-ups. These three annual assessments will be conducted electronically via the ProHEAD online platform in the computer rooms of the respective schools (for details on the instruments used at the school-based assessments, see the study protocols of the other ProHEAD sub-projects in this issue [29–32]). Based on the screening results, each participant will be allocated to one of the five ProHEAD RCTs. Participants who meet eligibility criteria for more than one RCT will be randomly allocated to one of the sub-projects.

**Fig. 1:**
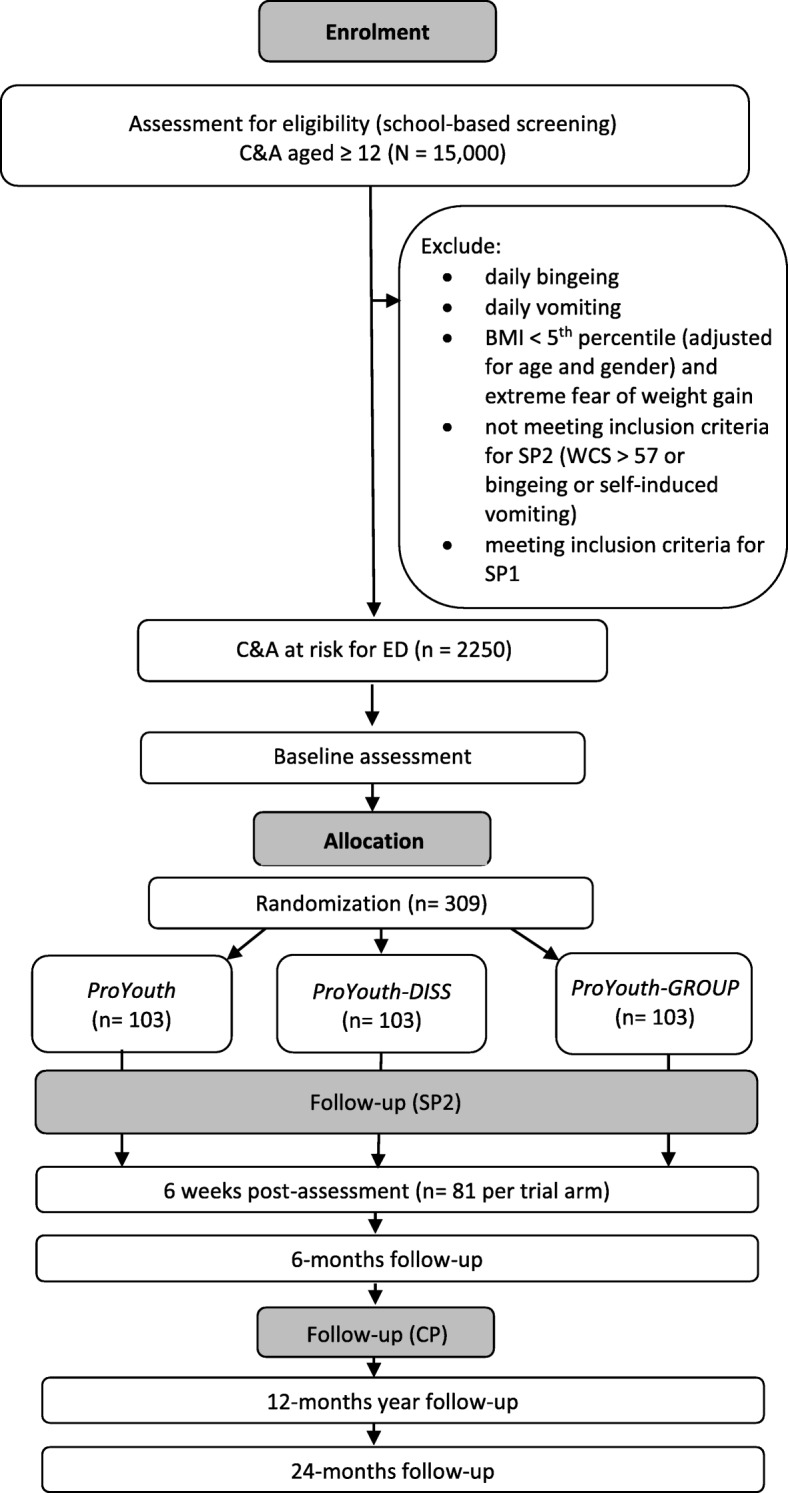
Participant flow diagram. Notes: *C&A* children and adolescents, *BMI* body mass index, *WCS* Weight Concerns Scale, *SP2* sub-project 2, *SP1* sub-project 1 [29], *CP* ProHEAD central project

Participants assigned to the present ED trial will receive an email inviting them to register for the study. In order to enroll, they will need to click on a link in this email to activate their user account and to complete a trial-specific baseline online assessment. Upon completion of this assessment, they will be randomized to one of the three study arms and then have access to the respective intervention (ProYouth, ProYouth-DISS, or ProYouth-GROUP) for a duration of 6 weeks. Trial-specific post and follow-up assessments will be conducted online 6 weeks after randomization and 6 months later (Fig. 1).

Participants who complete the post-assessment after 6 weeks (primary outcome) will receive an online gift voucher (20€).

There is no obvious risk for participating C&A. Participation in the trial does not restrict utilization of conventional healthcare (e.g., counseling, treatment, medication), and information on where to seek help in case of ED-related and/or mental health problems will be provided for all participants. Use of standard healthcare services will be assessed in detail as part of the 12 and 24 months follow-ups in the school-based assessments of the ProHEAD CP.

**Fig. 2:**
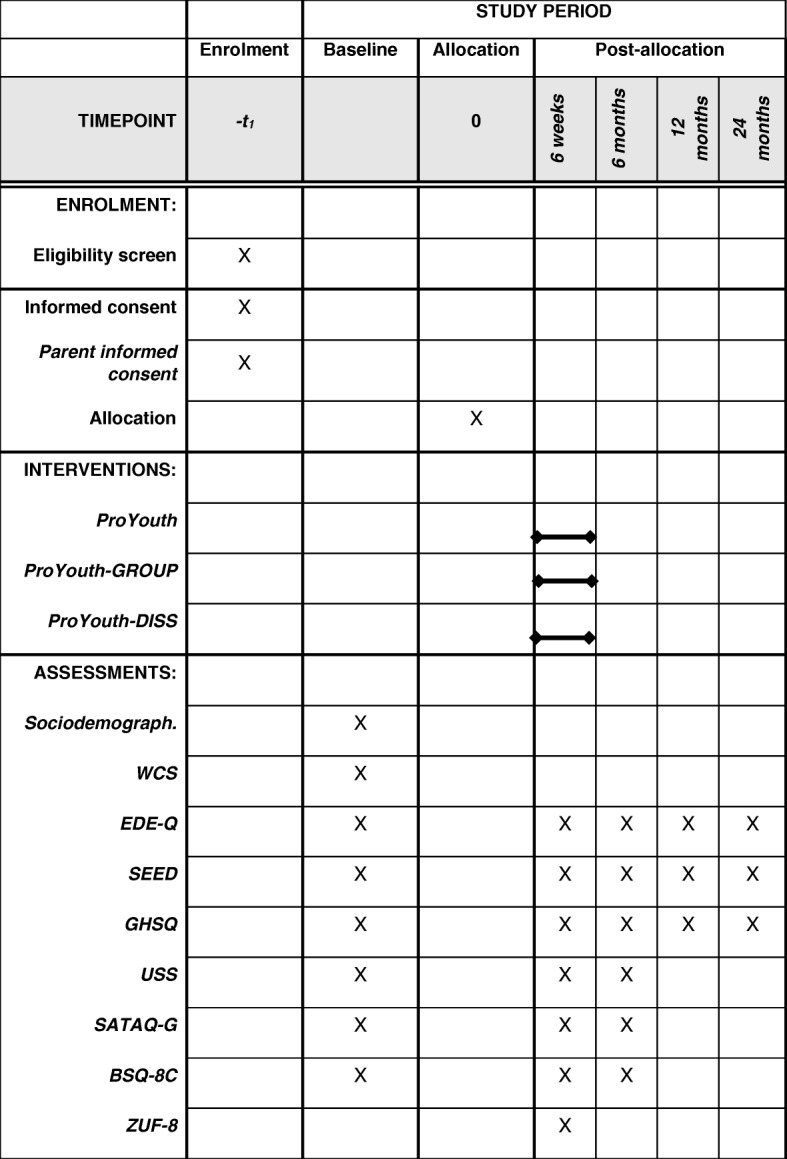
Schedule of enrolment, interventions, and assessments. Notes: –*t1* prior to participation, *Sociodemograph.* sociodemographic characteristics, *WCS* Weight Concerns Scale, *EDE-Q* (Child version of the) Eating Disorder Examination-Questionnaire, *SEED* Short Evaluation of Eating Disorders, *GHSQ* General Help-Seeking Questionnaire, *USS* Universal Stigma Scale, *SATAQ-G* German version of the Sociocultural Attitudes Towards Appearance Questionnaire, *BSQ-8C* 8-item version of the Body Shape Questionnaire, *ZUF-8* German version of the Client Satisfaction Questionnaire

Figure 2 shows the schedule of enrolment, interventions, and assessments of the present trial in detail. The recommended Standard Protocol Items: Recommendations for Interventional Trials (SPIRIT) 2013 checklist with items to address in clinical trial protocols is provided as Additional file 1.

### Randomization and blinding

Eligible C&A will be randomized to one of the three interventions based on an allocation sequence derived from a computerized random number generator at a 1:1:1 ratio. The allocation scheme is embedded into the ProHEAD online platform; i.e., participants are automatically assigned to one of the study arms and informed electronically about the outcome without researchers having access to the allocation scheme. Randomization will be stratified by gender. Due to the study characteristics, blinding of participants and/or online counselors is not possible because participants will be informed as to which intervention they were assigned, and counselors need to provide guidance accordingly.

### Interventions

#### ProYouth

Participants randomized to the control condition will participate in ProYouth [41] for 6 weeks. ProYouth is an Internet-based intervention for the prevention and early intervention of EDs. It consists of modules with different intensities, and participants can utilize the program in accordance with their individual needs and preferences. ProYouth includes a comprehensive section on psychoeducation, a forum for peer-to-peer support, and optional online chats with a psychologist. In addition, ProYouth includes a monitoring and feedback system; i.e., once a week participants receive an email invitation to answer a short online questionnaire on their Smartphone or PC and directly receive personalized feedback on their current status and the development of ED-related attitudes, behaviors, and symptoms over time. In case participants develop severe ED symptomatology during their participation, a ProYouth counselor automatically receives a notification via email. The counselor then contacts the participant personally and invites her/him to an individual chat session in order to clarify the need for further support. If needed, the counselor offers the participant assistance in seeking professional face-to-face help.

#### ProYouth-DISS

A structured dissonance-based intervention will be provided as an add-on to ProYouth; i.e., participants will have access to an enhanced version of ProYouth for 6 weeks. The ProYouth-DISS intervention aims at reducing ED risk factors by inducing cognitive dissonance. This approach assumes that the thin ideal and thin-ideal internalization play a major role in the development of ED. Internalized thin ideals in combination with social comparison processes result in increased body dissatisfaction. High levels of body dissatisfaction can cause dieting, restrained eating, and other compensatory measures to reduce body weight. This can be considered one of the developmental pathways of ED. ProYouth-DISS aims at counteracting these processes by influencing the internalized thin ideal and other weight- and shape-related beliefs and attitudes. As part of the intervention, participants will be asked to actively criticize such dysfunctional attitudes and beliefs. The discrepancy between the participants’ own beliefs and attitudes and the position they are representing in this situation causes dissonance. Because of the human tendency to seek consistency between attitudes and behaviors, this dissonance is reduced by a change of beliefs and attitudes (e.g., thin-ideal internalization, body dissatisfaction). Female participants will learn to challenge the thin ideal. In addition, they will be asked to engage in exercises aimed at reducing body image concerns. Male participants have access to an adapted version of ProYouth-DISS that challenges the muscular body ideal [42]. Videos with peers guide participants through the program and motivate them to attend the sessions. Participants work through the materials individually and may interact with a counselor on demand. The module consists of six sessions. As part of the weekly ProYouth monitoring emails, participants will be reminded to complete the ProYouth-DISS sessions. In addition to the structured ProYouth-DISS module, participants are encouraged to use the standard ProYouth modules.

#### ProYouth-GROUP

A clinician-guided CBT group intervention will be provided as an add-on to ProYouth. The preliminary efficacy of CBT interventions in terms of reduction of body image problems and ED symptoms has been confirmed in both face-to-face and online settings [27]. Based on this evidence, the ProYouth-GROUP intervention will use a CBT approach to address factors that contribute to the development and maintenance of body dissatisfaction, weight/shape concerns, and disordered eating. A specific focus will be on environmental risk factors such as stigmatization and the perceived pressure from peers and media to conform to social appearance ideals. This pressure is assumed to increase both the internalization of social appearance ideals and body comparison tendencies, which subsequently lead to an increase of body dissatisfaction, weight/shape concerns, and disordered eating. Therefore, the ProYouth-GROUP intervention will teach skills for understanding and counteracting such pressures from peers and media and convey ways how to normalize one’s own eating behavior. The group chats focus on topics such as media influences on body dissatisfaction, strategies to improve self-esteem and body image, and emotion regulation skills. In addition, participants are asked to engage in cognitive behavioral exercises following each session. The last session will highlight the importance of seeking help early, discuss barriers to help-seeking, and illustrate strategies on where and how to seek professional help. The intervention consists of six 90-min group chat sessions facilitated by a qualified psychologist. Separate groups will be established for female and male participants. Videos with peers guide participants through the program and motivate them to attend the chat sessions. They are also reminded to join the chat sessions as part of the weekly ProYouth monitoring emails. In addition to this structured module, participants are encouraged to use the standard ProYouth modules.

### Assessments, measures, and outcomes

Three assessments are conducted at participants’ high schools (i.e., screening, 12 months follow-up, and 24 months follow-up) as part of the CP of the ProHEAD Consortium. Additional trial-specific assessments are conducted prior to randomization (baseline), at the end of the intervention (after 6 weeks), and at 6 months follow-up (Fig. 2). All assessments are based on self-report and conducted online via the ProHEAD platform.

### Screening

ED-related eligibility criteria will be checked via two screening instruments. The Weight Concerns Scale (WCS) is a one-dimensional instrument consisting of five Likert items that assesses concerns about body weight. A WCS score > 57 is associated with an elevated risk for developing an ED [33, 34]. The Short Evaluation of Eating Disorders (SEED; [35]) assesses body weight and height used to calculate the BMI as well as key ED symptoms (fear of weight gain, distortion of body perception, over-concern with weight and shape, frequency of binge eating, frequency of compensatory behaviors) over the past 4 weeks.

### Primary outcome

The primary outcome of the present trial is ED symptomatology at the end of the intervention assessed with the child version of the Eating Disorder Examination-Questionnaire (ChEDE-Q; [43]). The ChEDE-Q measures disordered eating over a 28-day period and includes 28 items and four subscales (Eating Concern, Shape Concern, Weight Concern, and Dietary Restraint) as well as a global score, which is an average of the subscales.

### Secondary outcomes

Secondary outcomes include ED symptomatology at 6, 12, and 24 months follow-up measured with the ChEDE-Q and the SEED. Additional outcomes are body dissatisfaction, appearance ideal internalization, ED-related stigma, and ED-related help-seeking at the end of the intervention and at 6 months follow-up. Body dissatisfaction is measured with the 8-item version of the widely used Body Shape Questionnaire (BSQ; [44, 45]). Research on the brief version (BSQ-8C) demonstrated good psychometric properties and sensitivity to change [46]. Items are rated on a 6-point Likert scale from 1 = “never” to 6 = “always”, with higher scores indicating higher levels of body dissatisfaction. Appearance ideal internalization is assessed using the internalization subscale of the German version of the Sociocultural Attitudes Towards Appearance Questionnaire (SATAQ-G) for female and male participants, respectively [47]. It consists of 6 items reflecting the adoption of the thin or muscular ideal as a personal standard. Stigma is measured with the ED-specific version of the Universal Stigma Scale (USS; [48]). The USS is an 11-item instrument. It consists of two subscales (impairment/distrust, blame/personal responsibility), with internal consistencies between 0.72 and 0.83 across different samples [48]. ED-related help-seeking is measured with an ED-specific version of the General Help-Seeking Questionnaire (GHSQ; [49]), a frequently used measure for help-seeking for psychological complaints. Acceptance of the online interventions (ProYouth, ProYouth-DISS, ProYouth GROUP) will be assessed with the German version of the Client Satisfaction Questionnaire (ZUF-8; [50]) adapted for the online setting. The ZUF-8 measures participants’ satisfaction with the intervention. Finally, compliance with the interventions and utilization of the various modules will be analyzed based on automatically recorded server logs.

### Health economic evaluation

The health economic evaluation will include an analysis of the cost-effectiveness and cost-utility of the interventions. In addition to the costs of the interventions, health-related quality of life and healthcare utilization will be assessed at 12 months follow-up using the KIDSCREEN-10 [51] and the Mannheimer Modul Ressourcenverbrauch (MRV; [52]), respectively. The KIDSCREEN-10 is a widely used 10-item instrument for the assessment of health-related quality of life. The MRV assesses detailed information on utilization of treatment for any kind of health problem (inpatient care, outpatient care, medication) within the past 12 months.

### Sample size calculation

We assume a small to medium effect (superiority of the two intervention groups over the control condition) on ED symptomatology. Repeated measures analysis of variance (ANOVA) will be conducted (time*group interaction, alpha = 5%). Assuming an effect of *f* = 0.15, a dropout rate of 20%, and correlations of 0.1 among repeated measures, 309 subjects need to be recruited (103 per group) to test the global hypothesis with 90% power. In case of significant findings for the main effect of study group, the proposed sample size will allow for post hoc pairwise group comparisons at a power above 80%.

### Statistical analysis

Primary data analyses will be based on the intention-to-treat (ITT) population; i.e., all participants randomized to one of the three study conditions will be included in the primary outcome analysis. Missing data patterns will be explored. Multiple imputations will be applied to impute missing data.

Repeated measures ANOVA will be conducted (time*group interaction, alpha = 5%) to test the primary hypothesis, i.e., differences in efficacy between the interventions. For the primary outcome, only the baseline and the post assessment after 6 weeks will be considered. In case of statistically significant differences, post hoc pairwise group comparisons will be conducted (Bonferroni correction). Repeated measures ANOVA will also be conducted to determine the effect of the interventions on metric secondary outcomes. Post-intervention symptom courses will be analyzed applying mixed effects models. In order to investigate gender-specific efficacy, multifactor ANOVAs will be conducted. Fisher’s exact test will be applied to compare actual help-seeking between the groups.

For the health economic evaluation, data on the costs of interventions and efficacy will be used to determine the incremental cost-effectiveness ratio (ICER) of the interventions. The ICER will provide the additional costs in monetary terms of the respective programs to be spent for gaining an additional outcome unit. Cost-utility analyses will provide information on cost per quality-adjusted life years (QALYs) gained by the various interventions. Bootstrapping procedures will be applied to provide cost-effectiveness planes and acceptability curves.

### Data management

The Coordination Center for Clinical Trials (KKS) Heidelberg will monitor study-related procedures at the five recruiting centers. Specifically, the recruitment of schools within the target regions and the recruitment of students within these schools will be monitored in order to ensure adherence to the study manual and documentation guidelines as well as equivalent procedures at all sites. In addition, a Data Safety and Monitoring Board (DSMB) will be established.

All study-related data will be stored on secure servers at the Principal Investigator’s institution for 10 years. Data transfer and storage will be encrypted. Data access is password-protected. Only approved study staff can access the data; after completion of the study, only investigators can access data. All data are based on self-report and will be assessed online through the ProHEAD platform, which ensures high data quality and integrity. Automated full and incremental backups will be conducted at regular intervals according to a predefined schedule. Data collection, handling, access, and backup procedures will comply with German and European legal regulations with respect to data protection and data security. Results of the study will be presented at international conferences and published in peer-reviewed journals. In addition, the main results of the consortium will be published on the project’s webpage (www.prohead.de) in order to inform participants.

## Discussion

In light of the high burden of illness and the economic and social costs associated with EDs, the need for efficacious and cost-effective ED prevention programs is evident. Internet-based delivery of such programs is increasingly being recommended in order to enhance the reach and scalability of interventions. The present trial is innovative in that it combines the advantages of online intervention delivery, the flexible intervention strategy of the existing program ProYouth, and the potential of two new structured additional modules developed based on the latest evidence in ED prevention research [11, 12]. These modules incorporate a dissonance-based approach and a CBT-based approach, respectively.

The inclusion of both female and male participants and younger age groups, and the use of an active control condition and long-term follow-up assessments represent further strengths of the study. The trial largely benefits from being conducted as part of the overall ProHEAD Consortium, which will ensure recruitment of a large representative school-based sample of C&A.

Importantly, we will conduct a comprehensive health economic evaluation in addition to the efficacy study. To date, only very few studies have investigated the costs and the cost-effectiveness of ED prevention programs, and the findings so far appear to be inconsistent [53, 54]. However, health economic evaluation is key for decision-making in mental healthcare and for implementation of new interventions in healthcare routines and health insurance reimbursement schemes.

Potential limitations and challenges of the present trial include the fact that uptake of and compliance with the interventions are unknown. Research on e-mental health in general has shown that rates of both intervention dropout and study dropout may be high in such trials, and that incomplete follow-up data often question the robustness of findings even when ITT analyses are conducted. Challenges with respect to recruitment and adherence have also been reported for ED-specific prevention efforts [55]. The inclusion of male participants in the trial also represents a challenge, as there is almost no research on ED prevention in male samples. However, given that EDs also affect boys and men, it is highly relevant to study interventions in male populations. In the present trial, the dissonance-based and CBT-based modules include specific contents for male participants. Finally, it is to note that the planned sample size will be too small to directly compare the efficacy of ProYouth-DISS and ProYouth-GROUP. Analyses on the relative efficacy will thus be explorative in nature.

In summary, we expect the present trial to make an important contribution to research in the ED field by informing us on the efficacy and cost-effectiveness of the newly developed prevention tools. In case of positive results, the nature of the interventions (e.g., the combination of automated and personalized components) and the availability of the technical infrastructure as part of the ProHEAD Consortium would allow for a broad dissemination and long-term sustainability of the interventions.

## Trial status

The trial was registered at the German Register for Clinical Trials (DRKS; DRKS00014679; https://www.drks.de/drks_web/navigate.do?navigationId=trial.HTML&TRIAL_ID=DRKS00014679). Recruitment for the present trial has started in November 2018.

## Additional file

Additional file 1: Standard Protocol Items: Recommendations for Interventional Trials (SPIRIT) checklist. (DOCX 49 kb)

## Abbreviations

AUDIT: Alcohol Use Disorders Identification Test
BMBF: Federal Ministry of Education and Research
BMI: Body mass index
BSQ: Body Shape Questionnaire
BSQ-8C: 8-item version of the Body Shape Questionnaire
C&A: Children and adolescents
CBT: Cognitive behavioral therapy
ChEDE-Q: Child version of the Eating Disorder Examination-Questionnaire
CP: Central project
DRKS: German Clinical Trials Register
DSMB: Data Safety and Monitoring Board
ED: Eating disorder
EU: European Union
GHSQ: General Help-Seeking Questionnaire
ICER: Incremental cost-effectiveness ratio
ICH-GCP: Guidelines for Good Clinical Practice
ITT: Intention-to-treat
KIDSCREEN-10: Health-related quality of life measure for children and adolescents
KKS: Coordination Center for Clinical Trials
MRV: Mannheimer Modul Ressourcenverbrauch
PHQ-A: Patient Health Questionnaire-9 modified for Adolescents
ProHEAD: Promoting Help-seeking using E-technology for ADolescents
QALY: Quality-adjusted life year
RCT: Randomized controlled trial
SATAQ-G: German version of the Sociocultural Attitudes Towards Appearance Questionnaire
SDQ: Strengths and Difficulties Questionnaire
SEED: Short Evaluation of Eating Disorders
SP: Sub-project
USS: Universal Stigma Scale
WCS: Weight Concerns Scale
ZUF-8: German version of the Client Satisfaction Questionnaire
